# Cerebellar circuits anticipate dopamine rewards

**DOI:** 10.64898/2026.02.02.703324

**Published:** 2026-02-02

**Authors:** Benjamin A. Filio, Amma Otchere, Subhiksha Srinivasan, Srijan Thota, Luke Drake, Lizmaylin Ramos, Philipp Maurus, Mark J. Wagner

**Affiliations:** 1 National Institute of Neurological Disorders & Stroke, National Institutes of Health, Bethesda, MD 20894, USA.; 2 Department of Neuroscience, Brown University, Providence, RI 02906, USA.

## Abstract

Predicting rewards is critical for learning. Although the cerebellum predicts rewards like water and food, it also famously coordinates physical actions like drinking and eating. To disentangle reward prediction from the movements to consume reward, we trained mice to push for delayed dopamine rewards delivered directly into the brain. Via two-photon imaging, we found that many cerebellar granule cells (GrCs) anticipated dopamine with sustained activity that terminated at reward delivery. GrC activity also “stretched” to match 1- or 2-s intervals before dopamine, thereby linking the action to the expected reward time. By contrast, most cerebellar climbing fibers (CFs) spiked just after dopamine delivery. GrC-CF activity also generalized between dopamine versus water rewards, both in individual mice and many individual neurons. Finally, naïve mice ‘rewarded’ only with CF stimulation also learned to execute a modest number of pushing movements, with similar predictive GrC activity. Thus, cerebellar circuits help animals learn to anticipate rewards even when no consummatory action is needed, suggesting deeper cerebellar integration in brain reward prediction networks.

## INTRODUCTION

Learning which actions yield desired outcomes involves reinforcing rewarded behaviors^3,4^—an important brain-wide process with key contributions from nigrostriatal circuits in which midbrain dopamine (DA) reward signals drive downstream associative learning in the striatum^5^. By contrast, the cerebellum has long been known to contribute to learning predictions^6–8^, such as near-simultaneous associations vital for sensorimotor functions^9–12^. Recent cerebellar studies have broadened the concept of prediction learning to include, among other things, “reward prediction.” In thirsty animals seeking water, various cerebellar neurons convey signals that could be useful for predicting water reward occurrence, estimating its timing, or associating it with actions or events^13–15^. On the other hand, extensive motor programs are required to “consume” physical rewards, like drinking water, eating food, and social interaction—a major confound given the cerebellum’s famous role in refining physical actions. Thus, although GrCs exhibit a variety of activity patterns predictive of water reward^16,17^, these signals might serve mainly to support water-associated actions, such as anticipatory licking or orofacial consummatory movements. Similarly, although CFs convey heterogenous reinforcement signals^18–24^, with transient spiking at water delivery or predictive cues or actions, these signals might instead help reinforce or refine water drinking movements. Similar caveats confound cerebellar contributions to other ethological physical rewards like food^25,26^ and socialization^27^. Studies have also found that cerebellar Purkinje cells^16,28^ and cerebellar nuclear neurons^29,30^ convey reward-predicting signals, but these too employed rewards requiring physical consumption.

Together, these results raise a critical question: does the cerebellum predict rewards per se, or only when actions are required to sense or consume them? If cerebellar circuits also predict rewards that do not need physical consumption, they may be more deeply integrated with broader brain reward prediction networks and their associated disorders like compulsive reward-seeking and substance use. To adjudicate these possibilities, we trained mice to push a robotic handle for delayed self-stimulation dopamine rewards that are delivered directly into the brain and thus passively consumed. Using two-photon imaging, we found that both cerebellar input streams, GrCs and CFs, predicted and encoded dopamine rewards. Numerous GrCs ramped activity up and down at varied times prior to dopamine, linking the action to the expected reward time. This activity temporally scaled to match 1- or 2-s delays. Dopamine delivery itself triggered widespread CF spiking. These patterns generalized between water and dopamine rewards, and thus likely encoded abstract features of rewarded behavior. Finally, providing only delayed optogenetic CF ‘rewards’ also drove naïve mice to push at modest rates. Together, these data demonstrate that the cerebellum helps anticipate rewarding outcomes even when no consummatory actions are required, broadening possible roles for cerebellar reward prediction.

## RESULTS

### Push-for-delayed-self-stimulation tasks compatible with two-photon cerebellar imaging

**Figure 1 |:**
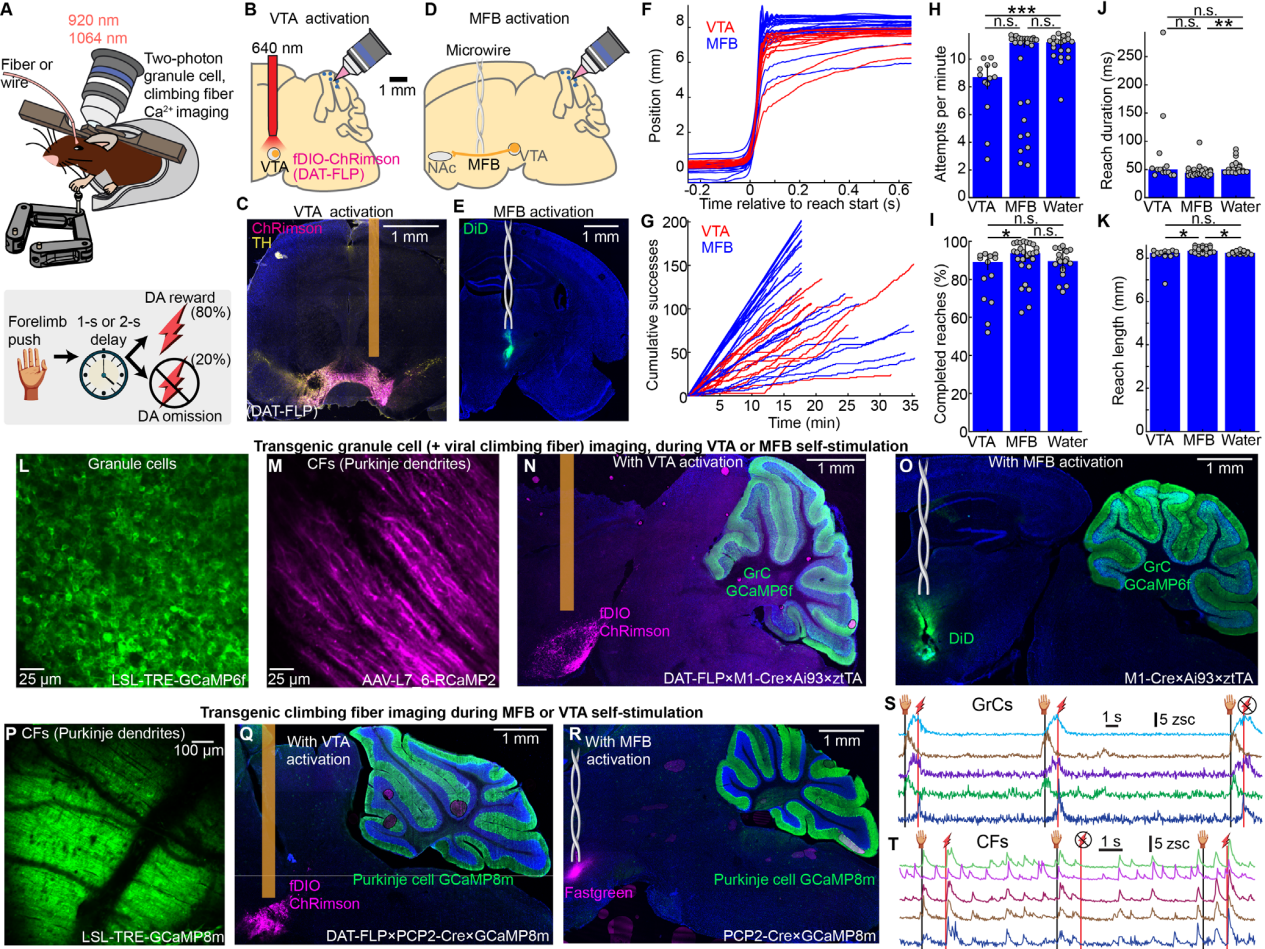
Push-for-delayed-self-stimulation tasks and simultaneous two-photon cerebellar imaging **A-K**, Behavior. **(A)** Task illustration. Head-fixed mice self-initiated right forelimb pushes of a robotic handle (max-8 mm extent) for delayed self-stimulation rewards. **(B,C)** For some mice (*n*=8), rewards were optogenetic activation of VTA dopamine neurons via FLP-dependent ChRimson (DAT-FLP mice; 2- or 5-ms pulses at 50 Hz for 250 or 500 ms, 10–15 mW of 640 nm laser power). **(D,E)** Alternatively, other mice (*n*=19) received microwire electrical stimulation of the medial forebrain bundle (5-ms 5-V pulses at 100 Hz for 250 ms). **(F)** Per-session average handle motion paths (13/8 VTA and 27/19 MFB sessions/mice). **(G)** Per-session cumulative successful reaches over time. **(H**–**K)** Reach attempts (**H**), the percentage of attempts that were completed (**I**), and for successful reaches, duration in ms (**J**) and extent in mm (**K**). Dots show each session’s median across trials. These and subsequent bars show median±median-absolute-deviation of the sample (m.a.d.) (**H–K** Bonferroni-corrected p= 0.1/1/7×10^−5^; 0.03/0.5/0.5; 0.4/0.004/1; 0.01/0.01/1 for VTA v MFB/MFB v Water/VTA v Water; 19/15 water sessions/mice). **L-T**, Two-photon imaging during push-for-dopamine. **(L)** *In vivo* two-photon GrC image, via Math1-Cre×LSL-tTA×LSL-TRE-GCaMP6f (20 mice). **(M)** *In vivo* two-photon image of Purkinje dendrites (reporting CF activity), via viral RCaMP2 in GrC imaging mice (5 mice). **(N)** To image GrCs during push-for-VTA optogenetic activation, we used DAT-FLP×M1-Cre×ztTA×GC6f transgenics (6 mice). We injected fDIO-ChRimson in VTA and implanted a fiber cannula (200 μm core, 0.22 NA). **(O)** Alternatively, to image GrCs during push-for-MFB stimulation, we implanted transgenics with a microwire cannula (DiD staining; 14 mice). **(P)** *In vivo* two-photon image of transgenic GCaMP8m in Purkinje dendrites (PCP2-Cre×LSL-GCaMP8m; 6 mice). **(Q)** For transgenic imaging of CF activity during VTA DA stimulation, we used DAT-FLP×PCP2×GC8 (2 mice). **(R)** For transgenic imaging of CF activity during push-for-MFB stimulation, we implanted microwire cannulas into PCP2×GC8 mice (4 mice). **(S,T)** Fluorescence over 3 trials from 5 example GrCs (**S**) or CFs (**T**). Icons indicate push and reward/omission times.

We sought an operant task where animals associate an action with a reward, with several features. First, animals should receive rewards without needing to “consume” them via physical movement—versus, e.g., water, which animals must drink and often begin licking for beforehand^31,32^. This helps avoid conflating neural representations of reward with those of consumption movements. Second, we temporally delayed the rewards so that transient neural representations of the operant action did not “mix” with later reward prediction signals. Third, to study GrC–CF dynamics, the paradigm would be surgically, genetically, and optically compatible with our two-photon cerebellar imaging strategy^16^. We thus designed a head-fixed push-for-delayed-self-stimulation task (Fig. 1A). We focused on two complementary self-stimulation rewards: (1) optogenetic activation of DA neurons in the ventral tegmental area^33–35^ (VTA) using ChRimson (Fig. 1B,C; Video S1) and (2) electrical activation of the medial forebrain bundle^36^ (MFB) (Fig. 1D,E, Video S2) (both left side only, contralateral to the reaching forelimb). VTA activation benefited from selectively targeting DA populations that signal rewarding outcomes^37^ (albeit heterogeneously^38^), but suffered from high viral-genetic and optical complexity (see **Imaging** below). By contrast, MFB electrical activation was simpler but less specific than VTA activation: while MFB activation yields DA release in the ventral striatum, it also produces rewarding or motivating effects through various non-dopaminergic fibers^39–42^. To obtain robust results, we studied both paradigms in parallel (MFB: 19 mice; VTA: 8 mice). Since findings were broadly consistent for both reward types, we hereafter write “DA reward” when referring to both.

**Extended Data Fig. 1 |:**
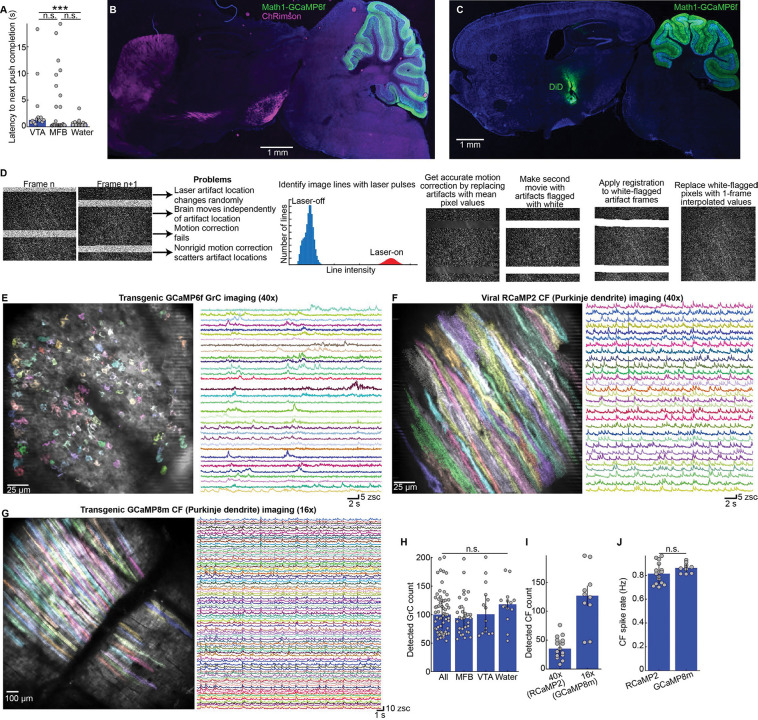
Related to Fig. 1 **A**, Latency to next reach completion defined as time between automatic robotic return of the handle from the previous trial and the end of the next pushing movement (p= 0.1/1/0.0006 for VTA vs MFB/MFB vs water/VTA vs water; 13 VTA, 27 MFB, and 19 water sessions). **B,C**, Larger histology images of GrC imaging + VTA surgery (**B**) or GrC imaging + MFB surgery (**C**). **D**, Strategy to temporally interpolate around optogenetic laser artifacts in the red channel for pulsed stimulation of the VTA. **E-G**, Example two-photon images and cell extraction results for GrC imaging (**E**) using cNMF^1^ and RCaMP-2-based (**F**) or GCaMP8-based (**G**) CF imaging and cell extraction, using PCA/ICA.^2^ **H-J**, Cell extraction counts across individual imaging sessions broken down by cell type and imaging condition. 61 GrC imaging sessions (33 MFB, 14 VTA, 14 water); 15 RCaMP-2 and 10 GCaMP8 CF imaging sessions. (**H**, p=0.4; **J**, p=0.2).

We trained animals to self-initiate right forepaw pushes of a robotic manipulandum^43^ (constrained to linear motion, maximum extent: 8-mm; Methods). Movements >6 mm in length were rewarded 1 s after completion with self-stimulation (VTA: 2- or 5-ms pulses at 50 Hz for 250 or 500 ms, 10–15 mW of 640 nm laser power; MFB: 5 ms, 5 V pulses at 100 Hz for 250 ms). After another 2-s delay, the handle began automatically returning to the start position over the next 2 s before animals were able to initiate the next trial. Training lasted 5–7 days. Expert mice typically made 100 or more successful reaches in each session (Figure 1F,G), attempting 8.7±0.2 and 11±0.1 trials per minute and succeeding 89±0.1% and 94±0.9% of the time, for VTA and MFB rewards respectively (Fig. 1H,I; 27/19 MFB and 13/8 VTA sessions/mice. All quantifications are median±median absolute deviation of the sample (m.a.d)). Successful reaches were 50±2 ms and 44±0.6 ms in duration and 8.3±0.02 mm and 8.4±0.02 mm in length for VTA and MFB respectively (Fig. 1J,K). After the robot returned the handle from the prior trial, mice completed the next push within 1.3±0.1 s (VTA) and 0.3±0.01 s (MFB; Extended Data Fig. 1A). For comparison, some mice were trained for another week to push for water, either before or after DA (15 mice, 5 DA-first). Across quantification metrics, MFB sessions exhibited comparable or higher motivation levels than water sessions, while VTA sessions exhibited comparable or lower motivation levels than MFB and/or water (Fig. 1H–K, Extended Data Fig. 1A). Overall, mice were motivated for self-stimulation and learned the task timing, allowing us to investigate whether and how cerebellar GrC-CF circuits predict and respond to DA rewards.

### Imaging cerebellar granule cell and climbing fiber activity during push-for-dopamine

To investigate DA prediction signals in the cerebellum, we focused on its two major input pathways—GrCs and CFs—which constrain downstream cerebellar computation. Recording many GrCs in a local microcircuit requires^44^ transgenic two-photon Ca^2+^ imaging. CF activity can be densely sampled via Ca^2+^ imaging of Purkinje dendrites, which faithfully reports complex spikes^45^ and thus CF input spikes^46^. Our GrC imaging (20 mice) employed 3 transgenes (Fig. 1L; Video S3; Math1-Cre×ztTA×GCaMP6f). For CFs (11 mice), we either used (1) viral RCaMP in Purkinje dendrites of GrC GCaMP transgenics (Fig. 1M; 5 mice; AAV-L7–6-RCaMP2); or (2) mice with transgenic GCaMP in Purkinje cells (Fig. 1P, Video S4, 6 mice; PCP2-Cre×GCaMP8m).

To combine imaging with push-for-VTA optogenetic activation required several additional workarounds. Optogenetically activating VTA requires driving opsin expression selectively in DA neurons. Since we already used cre to restrict cerebellar GCaMP expression, an orthogonal genetic driver was needed to restrict opsin to DA cells. We selected DAT-FLP^47^ to virally transfect VTA DA cells with FLP-dependent opsin (AAV-fDIO-ChRimson). Thus, we injected virus and implanted fibers in the VTA of crosses of DAT-FLP with either GrC GCaMP mice (Fig. 1N, Extended Data Fig. 1B, DAT-FLP×M1-Cre×ztTA×GC6f), or CF GCaMP mice (Fig. 1Q, DAT-FLP×PCP2-Cre×GC8m). Additionally, one-photon optogenetics spectrally interfered with the two-photon imaging red channel, which we mitigated (1) via optics (activating ChRimson at 640 nm plus extra emission filters), and (2) by temporally interpolating our RCaMP imaging data around the short 640 nm pulses (Extended Data Fig. 1D, Methods). Alternatively, to image during push-for-MFB electrical activation, we simply implanted microwire cannulae in either GrC (Fig. 1O, Extended Data Fig. 1C) or CF imaging transgenic animals (Fig. 1R). We sorted GrCs using nonnegative matrix factorization (cNMF^1^) and computed z-scored fluorescence (Fig. 1S, Extended Data Fig. 1E). We sorted Purkinje dendrites using independent components analysis (PCA/ICA^2^) (Fig. 1T, Extended Data Fig. 1F,G), detected spikes via deconvolution and thresholding, and computed spike rates over 150 ms windows. We imaged 100±3 GrCs, and 35±4 or 127±6 Purkinje dendrites per session with RCaMP or GCaMP respectively (61, 15, and 10 sessions, respectively, Extended Data Fig. 1H–J).

In prior studies, cerebellar water reward signals spanned several regions, including Lobule VI^16,17,48^. Across species and spatial scales, Lobule VI also encodes and contributes to a variety of affective functions^49–53^, as do its target output regions in the cerebellar nuclei^54^. To thoroughly characterize DA reward signals in a consistent cerebellar region, we therefore imaged the right vermis of lobule VI (ipsilateral to the reaching forelimb, contralateral to the self-stimulation sites). We performed imaging studies after ~1 week of training.

### Many granule cells ramped activity across the interval until expected dopamine reward

**Extended Data Fig. 2 |:**
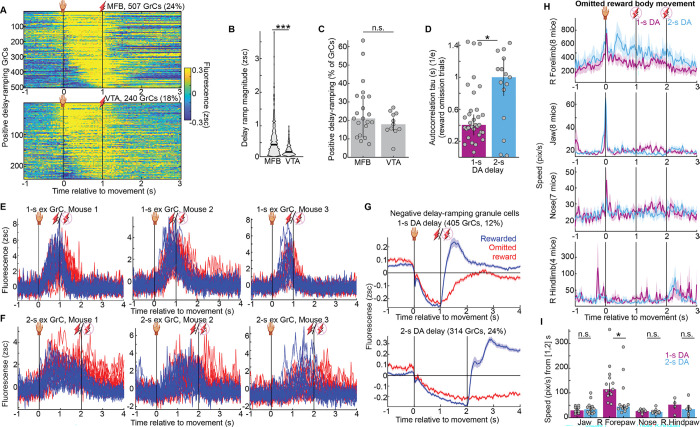
Related to Fig. 2 **A-C**, Comparison of imaging results during push-for-MFB sessions versus push-for-VTA activation sessions (6/11/240 VTA and 14/21/507 MFB mice/session/ramping GrCs), analogous to Figs. 2C,M (**B**, p<10^−6^; **C**, p=0.2). **D**, Single-session exponential fit time constants for the per-session mean-across-cells temporal autocorrelation plots shown in Fig. 2P (p=0.02, 32 and 15 sessions). **E,F**, More example GrCs, analogous to Fig. 2Q,R. **G**, Analogous to Fig. 2S, averages in rewarded and omission trials across all negative-ramping GrCs in 1- and 2-s delays (20 and 9 mice). **H,I**, Body tracking data by dlc from the subset of mice with high quality movement tracking in both 1- and 2-s recording sessions (showing only reward omission trials). 2-s sessions did not exhibit more (or exhibited less) body movement than 1-s sessions did during [1, 2] s (p=0.01; p=1; p=0.7; p=1), arguing against the longer 2-s GrC ramps resulting from extended uninstructed body movement. From left to right: p=1; 0.02; 0.3; 1. # of mice=8; 8; 7; 4. Session counts 1-s/2-s: 12/14; 13/13; 11/13; 4/7.

**Figure 2 |:**
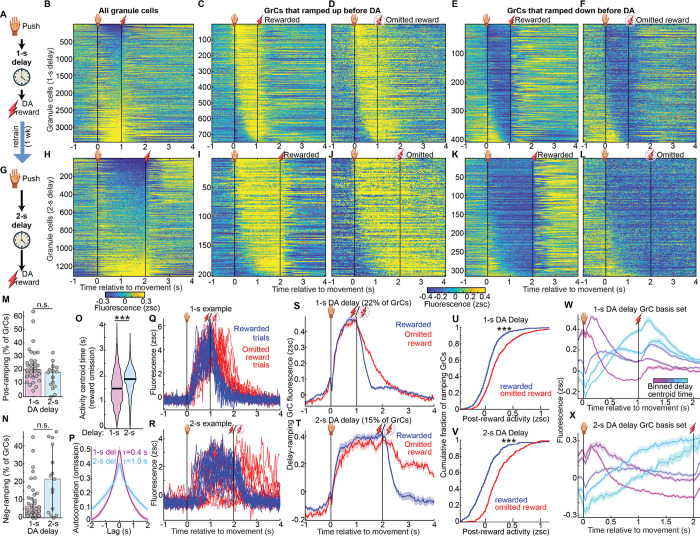
Granule cell activity ramps anticipate dopamine reward and temporally scale to match its expected timing **A-F**, Push for 1-s-delayed DA task. **(A**) Illustration. **(B)** Trial-averaged activity. Rows show individual neurons (3,464 cells, 20 mice). Here and in all subsequent panels “relative to movement” means movement endpoint if not otherwise specified. These and other vertical lines denote movement and reward. **(C,D)** 22% of GrCs (759) ramped up activity before DA, classified by mean activity from [0.9, 1] s >0.1 zsc and higher than before ([−0.3, 0] s) and after ([1.4, 1.7] s). Rasters computed for rewarded (**C**) or omitted reward (**D**) trials. **(E,F)** 12% of GrCs (405) ramped down before DA, classified inversely to **C,D**. **G–L**, 9 1-s-expert mice retrained with 2-s delays. Panels mirror **A–F** (1,310 GrCs; 15%(201)/24%(314) positive/neg. ramping). **M,N,** Per-session percentage of GrCs classified as positive (**M**, p=0.06) and negative delay-ramping (**N**, p=0.07; 32 1-s and 15 2-s delay sessions). **(O)** Across all GrCs, activity centroid times on omitted reward trials (timepoints with activity >0.1 zsc between [0, 4] s). For cells more strongly suppressed than activated, criteria were inverted (<−0.1 zsc; p<10^−6^). **(P)** For all GrCs, autocorrelation averaged first across GrCs and then across sessions, on omitted reward trials. Time constants were longer in 2-s delays (*τ*=1.0±0.06 s vs 0.41±0.02 s, p=0.02; Extended Data Fig. 2D). **(Q,R)** 15 rewarded and omitted reward trials from individual exemplar 1-s-delay (**Q**) and 2-s-delay (**R**) ramping GrCs. **(S,T)** Mean across positive ramping GrCs of the trial-averaged activity in 1- (**S**) or 2-s delay tasks (**T**; 759/20 and 201/9 GrCs/mice, respectively). **(U,V)** Cumulative histograms across ramping GrCs of activity after reward vs omitted reward in 1- (**U**) and 2-s (**V**) delays (p<10^−6^, mean activity [0.4,0.7] s after reward/omission). **(W,X)** All GrCs in 1-s or 2-s delay recordings binned by their delay period activity time centroids and averaged across cells per-bin, illustrating a temporally-scaled basis set (bin edges: [−0.1 0.2 0.4 0.55 0.65 0.98] s and [−0.1 0.4 0.8 1.2 1.6 1.95], cell counts per bin: [412 312 330 158 294] and [265 294 506 144 32]; for **W** and **X**, respectively).

To examine reward-predictive signals in GrCs, we computed the trial-averaged activity aligned to the end of the reaching movement for each GrC and sorted GrCs by activity level before reward (Fig. 2A,B). Many GrCs became more active during the delay between the push and the DA reward. Specifically, we classified 22% of GrCs as “ramping”, if fluorescence was >0.1 zsc just prior to reward and higher than the periods before or after (Fig. 2C,D; 100 ms before reward versus both [400, 700] ms after reward and 300 ms before movement). Inversely, we identified an additional 12% of GrCs that decreased their activity below baseline during the delay (Fig. 2E,F). Disaggregating the data by reward type, we found qualitatively similar results for VTA and MFB datasets, but with stronger ramping signals in MFB reward sessions (Extended Data Fig. 2A–C), possibly related to the higher motivation levels in the MFB task (Fig. 1, **Discussion**). Overall, many GrCs progressively increased or suppressed activity in anticipation of DA reward—despite that no physical action after the initial pushing movement was required to obtain or consume the DA reward.

### Dopamine-anticipating granule cell activity ramps temporally scaled to match expected reward timing

We hypothesized that GrC activity ramps “linked” the forelimb movement to the future expected reward. This predicted that longer intervals before DA reward would require longer GrC ramps. To test this possibility, for a subset of animals imaged on 1-s delays, we retrained for another week using a 2-s delay (Fig. 2G, Video S5, 9 mice). Visually, most GrC activity profiles appeared temporally “scaled” to the longer delay duration (Fig. 2H). Via the same criteria above, we identified 15% of GrCs that ramped activity upwards across the 2-s delay, again with varied kinetics (Fig. 2I,J), and 24% of GrCs that ramped activity downwards (Fig. 2K,L). Across individual 1-s and 2-s sessions, positive-ramping GrCs comprised 20±1% and 18±2%, while negative-ramping GrCs comprised 6.5±1% and 22±5% of cells, respectively (Fig. 2M,N, 32 1-s and 15 2-s delay sessions; **Discussion**).

We next quantified temporal scaling across the entire GrC population. Because delivering rewards at different times will directly drive different activity patterns, we restricted these analyses to omitted-reward trials, where animals experienced no external events after forelimb movement. First, we quantified each GrC by the centroid time of its elevated activity between [0, 4] s on omitted reward trials (Fig. 2O). GrC activity was centered substantially later in the trial in 2-s experts than in 1-s experts (p<10^−6^). Alternatively, we considered the temporal autocorrelation function as a metric of the characteristic timescale of GrC activity (Fig. 2P). We found that autocorrelation time constants were significantly longer on average in 2-s experts than in 1-s experts (Extended Data Fig. 2D, *τ*=1.0±0.06 s vs 0.41±0.02 s, p=0.02). To exclude an alternative interpretation, we tracked body movement and did not find any “temporally extended” actions in 2-s sessions compared to 1-s sessions (Extended Data Fig. 2H,I). GrC activity thus temporally scaled to match longer intervals before DA.

Suspecting that GrCs ramped for as long as animals continued to await DA reward, we more closely compared rewarded and omitted-reward trials. For both 1-s and 2-s delays; for both positive and negative-ramping GrCs; for individual neurons on single trials (Fig. 2Q,R, Extended Data Fig. 2E,F); and in trial-averages across the population (Fig. 2S–V, Extended Data Fig. 2G), activity ramps terminated more abruptly following reward delivery, but persisted longer after reward-omission, consistent with active maintenance of an expectant internal state. Alternatively, by binning the entire GrC population based on delay activity timing, we found that ascending and descending ramps of varying kinetics all roughly doubled in duration in 2-s vs 1-s delays (Fig. 2W,X). Thus, GrCs ramped up and down with heterogeneous profiles that temporally scaled to match the expected time of reward. GrC activity thereby temporally linked actions to expected DA rewards.

### GrC populations represented expectation of DA and water rewards similarly

**Extended Data Fig. 3 |:**
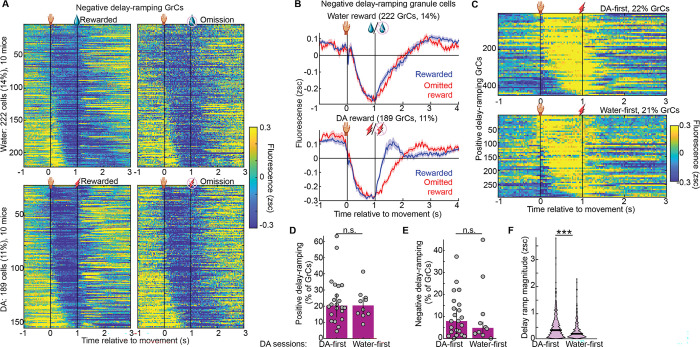
Related to Fig. 3 **A**, Negative-ramping GrC rasters in water and dopamine sessions from the same 10 mice, analogous to Fig. 3D–G. **B**, Average across negative ramping GrCs from A in water and dopamine. **C-F**, Comparison of dopamine-reward imaging sessions in mice pre-trained in water versus those not, analogous respectively to Figs. 2D,H,I,J. 22/452 DA-first and 10/295 water-first sessions/ramping GrCs, p values respectively 0.8, 0.7, <10^−6^, arguing that there is no trend for prior training with water reward to be either necessary or facilitating for subsequent dopamine-reward prediction signals.

**Figure 3 |:**
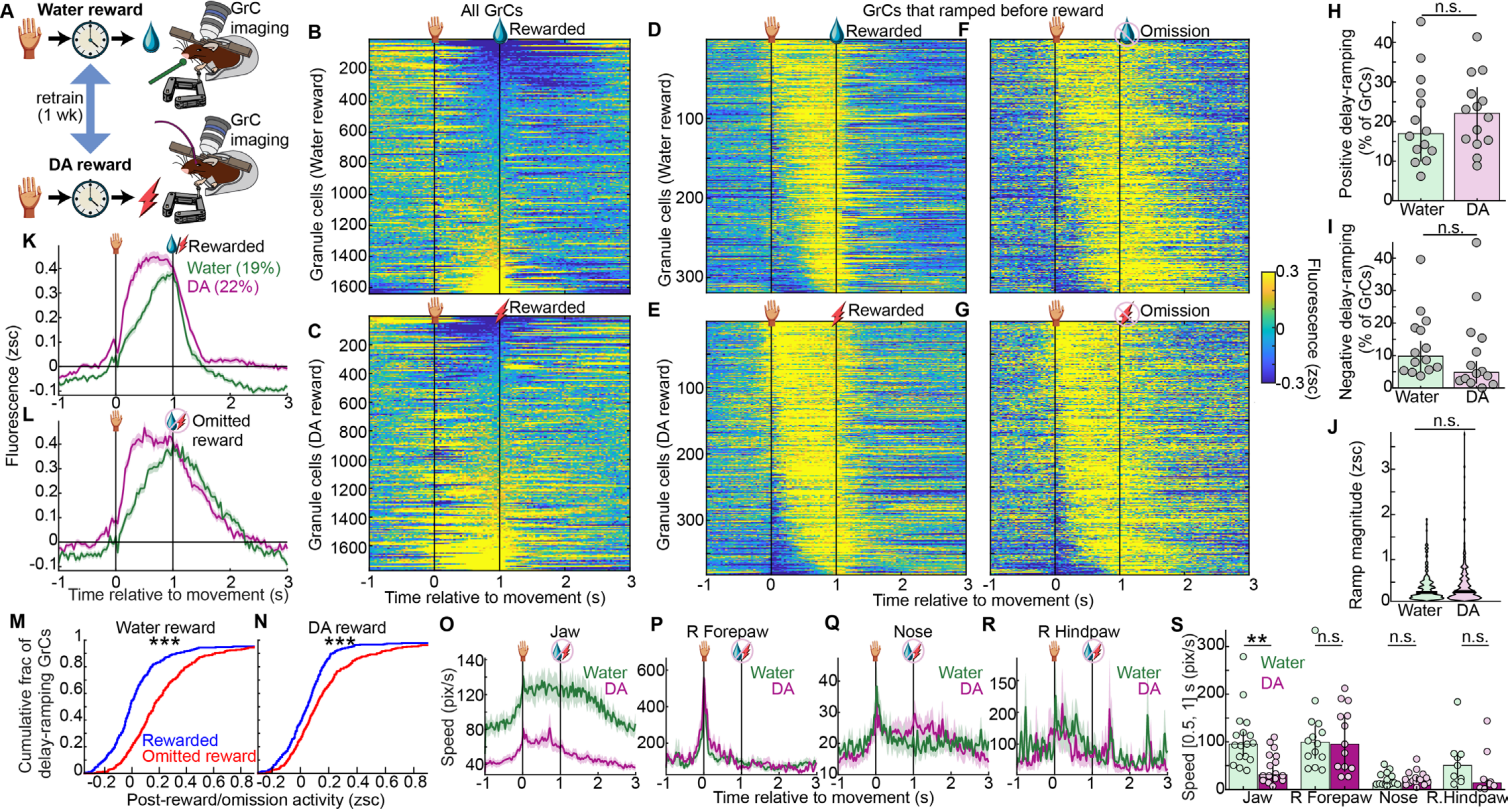
GrC populations represented expectation of DA and water rewards similarly **A,** GrCs imaged across push-for-water and push-for-DA tasks trained sequentially, 1 week each (10 mice, 6 water-first / 4 DA-first). **B,C,** For water (**B**) and DA (**C**) tasks, each row shows one GrC’s activity averaged across rewarded trials, sorted by activity just before reward ([0.9, 1] s; 1,640 GrCs in water and 1,774 GrCs in DA). Water and DA data sorted separately. **D–G**, GrCs that ramped up before reward (defined as in Fig. 2). Rasters show average activity across rewarded (**D,E**) or omitted reward (**F,G**) trials in water (**D,F**) or DA (**E,G**) tasks (319/382 water/DA GrCs, sorted by centroid of elevated activity timepoints [>0.1 zsc] during the delay). **H,I**, prevalence per session of positive (**H**, p=0.5) and negative (**I**, p=0.1) delay-ramping GrCs (14 DA and water sessions from 10 mice). **J**, Average pre-reward activity of ramping GrCs ([−0.1, 0] ms from reward; p=0.2, 319 water and 382 DA GrCs from 10 mice). **K,L**, Average activity of ramping GrCs. **M,N**, Histogram across ramping GrCs of post-reward vs post-omission activity ( [1.4, 1.7] s, both p<10^−6^). **O-S**, Body movement. (**O-R**) Average body coordinate speed across omitted reward trials. (**S**) Mean speed before reward [0.5 1] s. From left: p = 0.003, 1, 1, 0.6; # of mice: 11, 13, 12, 6. Session counts water/DA: 17/16; 14/13; 16/15;

Most prior studies of reward signaling in cerebellar circuits relied on water reward in thirsty animals—a reward that is both homeostatic and ethologically relevant, but complicated by extensive orofacial drinking movements. To determine the generality of GrC reward representations in individual animals, a subset of mice imaged in push-for-DA were also imaged in push-for-water (Fig. 3A, Video S6, 10 mice, 6 trained in water first, 4 in DA first). In both water and DA reward tasks, these mice exhibited substantial delay-period GrC activation (Fig. 3B,C). GrCs that ramped up activity across the delay comprised similar proportions of the population in DA (22%) and water (19%) reward contexts, both with a variety of temporal kinetics (Fig. 3D–G). Similarly, negative ramping cells comprised 15% and 19% of GrCs in DA and water, respectively (Extended Data Fig. 3A,B). Across individual imaging sessions, positive delay-ramping cells comprised 22±2% (DA) and 17±2% (water) of GrCs (Fig. 3H), while negative ramping cells comprised 5±1% (DA) and 10±1% (water) of GrCs (Fig. 3I). Activity levels of ramping GrCs just prior to DA and water were comparable (Fig. 3J). To exclude that prior water training facilitated subsequent DA prediction signals, we confirmed that activity in DA sessions was similar whether or not animals previously trained with water reward (Extended Data Fig. 3C–F). For both water and DA rewards, GrC ramps terminated more rapidly following reward than reward omission (Fig. 3K–N), suggesting that ramps persist while animals await reward, regardless of its type. In contrast to water reward which elicits licking both before and during reward consumption, no physical action is needed to consume the DA reward—yet animals might still compensate via new uninstructed anticipatory movements. To test this, we tracked body movement on omitted reward trials using DeepLabCut^55^ and confirmed that DA sessions elicited far less jaw movement (Fig. 3O), without adding other overt anticipatory body movements (Fig. 3P–S). Thus, GrCs linked the action to the future expected reward time via varied ramping profiles prior to both DA and water.

### Individual GrCs temporally scaled with DA delay duration and generalized across water and DA reward

**Extended Data Fig. 4 |:**
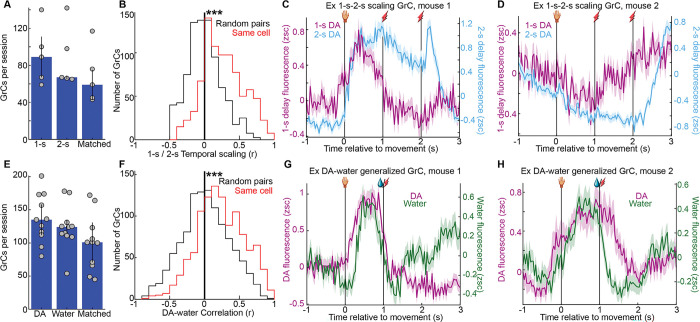
Related to Fig. 4 **A**, GrC counts in 1- and 2-s delay recordings, and the number of GrCs matched across both per session (5 sessions). **B**, Histogram from Fig. 4F, here compared to the distribution when cell identities were randomly shuffled (i.e., comparing a cell in one context to a randomly chosen cell from the other context), showing that correlations were substantially higher than predicted by chance (p<10^−6^). **C,D**, Additional example temporally-scaling GrCs analogous to Fig. 4H,I. **E-H**, Same as **A-D** but for dopamine v water generalization (11 sessions). **F**, p<10^−6^.

**Figure 4 |:**
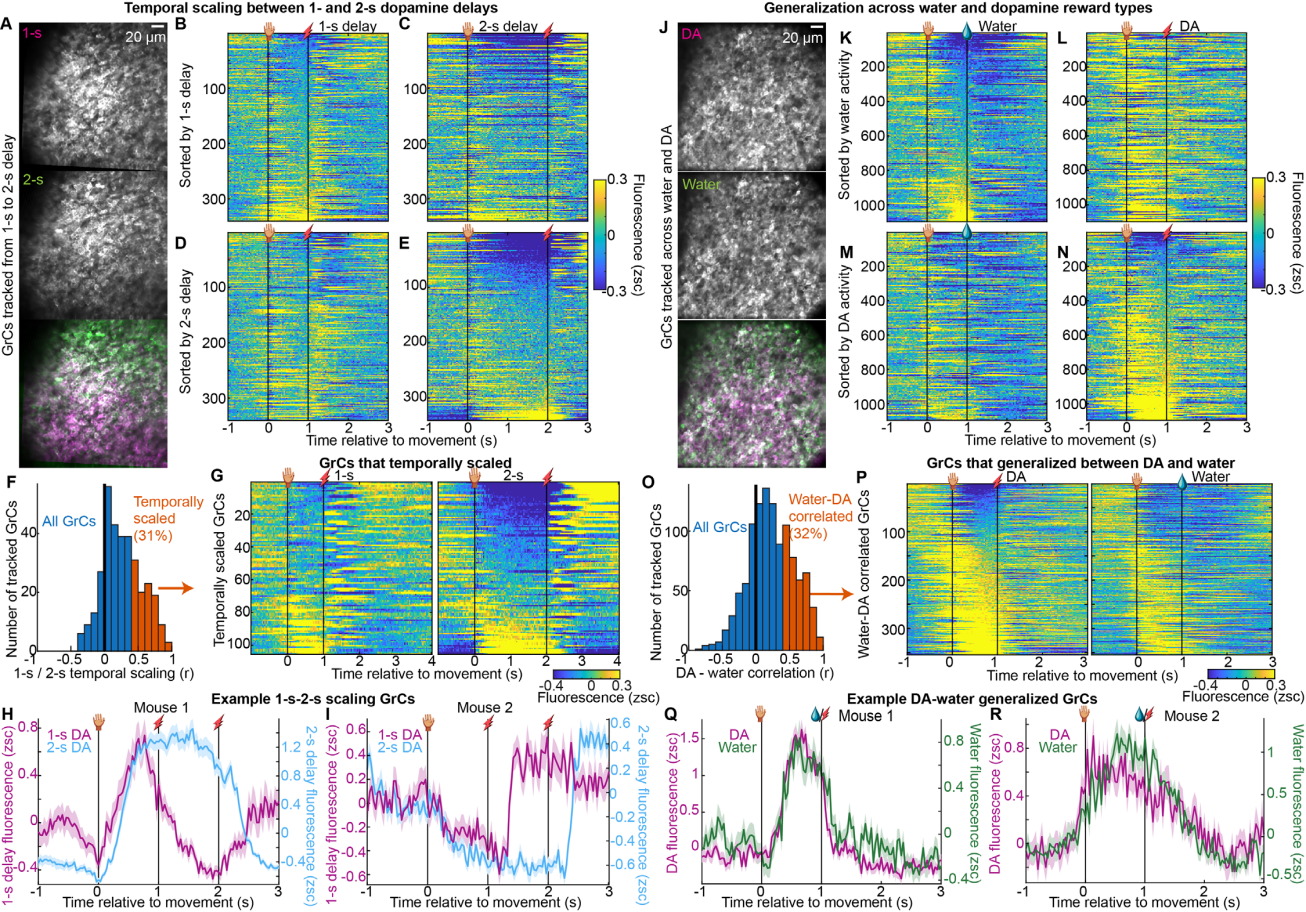
Many individual GrCs generalized across action-dopamine delay durations or reward types **A-G**, Individual GrCs tracked across 1-s and 2-s delays. **(A)** Same GrC imaging field in a 1-s- and 2-s-expert session 1 week apart. **(B-E)** Trial-averaged activity profiles on rewarded trials for all neurons tracked across 1- and 2-s-delay sessions (340 cells from 5 1-s/2-s-registered imaging fields in 4 mice). **(F)** We computed a “temporal scaling” index as the correlation coefficient (Pearson’s *r*) from [−1,3] s between a neuron’s 1-s-delay trial-averaged activity profile and a “compressed” version of its 2-s-delay profile (by temporally compressing the 2-s delay period into 1-s). We classified 31% of neurons as temporally scaled (*r* >0.4 and *p*<0.05). **(G)** Trial-averaged activity in 1-s and matched 2-s sessions for 105 temporally scaled GrCs. **H,I**, Example GrCs from **G** from two mice, showing mean±SEM across trials. **H-N**, Analogous to **A-F** for water-DA GrC registration. **(H)** An imaging field in a water and DA session. **(I-L)** Trial-averaged activity of GrCs tracked between water and DA reward sessions (1,098 GrCs from 11 water-DA-registered imaging fields in 8 mice). **(M)** Correlation coefficient (Pearson’s *r*) from [−1,1] s between each neuron’s activity profile in water reward with that in DA reward. We classified 32% of neurons as water-DA-correlated (*r* >0.4 and *p* < 0.05). **(N)** Trial-averaged activity of 354 water-DA-correlated GrCs in DA and water imaging sessions. **Q,R**, example cells from the group in **N**.

Having demonstrated a common GrC population representation across both delay durations and reward types, we questioned whether *individual* GrCs also generalized: either by temporally scaling across 1- and 2-s delays; or by cross-activating for water and DA. In some 2-s-delay recordings, we were able to register individual GrCs to recordings ~1 week prior with a 1-s delay (Fig. 4A). We then restricted analysis to GrCs matched across 1- and 2-s-delay recordings, and again generated trial-averaged raster plots (Fig. 4B–E, Extended Data Fig. 4A 340 GrCs from 5 imaging fields in 4 mice). Visually, only a fraction of cells had activity that seemed similar across delay durations. To identify individual GrCs with activity that temporally “stretched” to the delay duration, we compared each neuron’s 1-s-delay activity to a “temporally scaled” version of its 2-s-delay activity, by compressing the activity during the 2-s delay into a 1-s span, and then computing the Pearson correlation coefficient on the resulting activity traces (Fig. 4F; substantially higher than shuffled controls, Extended Data Fig. 4B). Setting cutoffs for correlation magnitude (r>0.4) and p-value (<0.05), we scored 31% of GrCs as temporally scaled (Fig. 4G). Some of these GrCs, for example, exhibited elevated activity during the 1-s delay that was stretched to match the longer delay in the 2-s-delay recording (Fig. 4G,H, Extended Data Fig. 4C). Other GrCs exhibited reduced activity during the 1-s delay that was extended into longer suppression in the 2-s-delay recording (Fig. 4G,I, Extended Data Fig. 4D). Thus, for many GrCs, activity profiles temporally scaled to the expected interval between action and DA reward time, despite no major associated changes in body movement (Extended Data Fig. 2H,I), suggesting that such cells represent reward temporal expectations.

We next examined whether individual GrCs played similar roles across both DA and water reward types. By registering neuronal identity across DA and water sessions (Fig. 4J), we matched 1,098 GrCs from 8 mice across both contexts (Extended Data Fig. 4E). When sorting these GrCs based on their activity level just prior to water (Fig. 4K,L) or DA (Fig. 4M,N), we observed only a subset of GrCs with similar activity profiles in both contexts. To identify neurons with cross-reward generalized activity, we computed for each GrC the correlation from [−1,1] s between its trial-averaged activity profile in water and that in DA (Fig. 4O, higher than shuffled controls, Extended Data Fig. 4F). The cutoffs applied for temporal scaling also classified 32% of GrCs with similar activity across DA and water recordings (Fig. 4P–R, Extended Data Fig. 4G,H). Thus, a subset of GrCs also generalized across reward types, despite the far greater orofacial movements in the water context (Fig. 3O), suggesting such cells may encode more abstract task features of reward-driven behavior.

### Many climbing fibers responded at short latency to dopamine reward delivery

**Figure 5 |:**
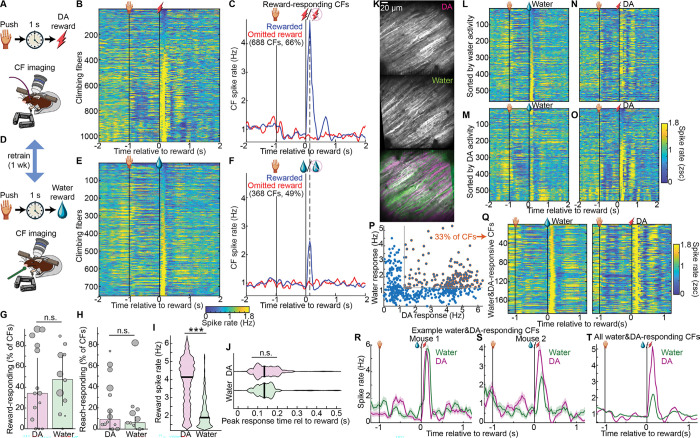
Dopamine rewards activated most CFs, many of which also spiked after water rewards **A-C**, Dopamine reward CF imaging. **(A)** Task illustration. **(B)** Average across rewarded trials of each CF’s activity aligned to reward delivery (1,048 CFs from 11 mice). **(C)** For 688 CFs (66%) classified as DA-responsive (spike rate [0.1,0.2] s post-reward >1.3 Hz), average across rewarded and omitted reward trials and grand-averaged across neurons (11 mice). **D-F**, Analogous to A-C for water reward (754 CFs from 8 mice, 368 CFs (49%) water-responsive). **G,H**, Per-session prevalence of reward-responding (**G**, p=0.8) and reach-responding CFs (**H**, p=0.6) (spike rate [−0.1,0.1] s relative to movement >1.3 Hz). Area of each session’s dot is proportional to number of CFs. **I,J**, Violin plot of each reward-responding CF’s spike rate [0.1,0.2]s (I, p<10^−6^, 688 DA and 368 water CFs) and peak response time (**J**, p=0.2). **K-T**, CF registration across water and DA recording sessions (**K**). **(L-O)** Tracked CFs sorted by water response **(L,N)** or DA response (**M,O**; 566 CFs from 8 mice). **(P)** Each cell’s mean activity [0.1 0.2] s from reward in a DA (x-axis) and water (y-axis) session. 33% of CFs were classified as reward-responsive in both contexts (>1.3 Hz). **(Q-T)** Trial-averaged activity of water & DA-responsive CFs in water and DA sessions, sorted by water responses (**Q**, 186 CFs) and as time-series plots from example cells from two mice (**R,S**, SEM across trials) and as an average across all such cells (**T**).

Synaptic integration of GrC inputs in Purkinje cells is dictated partly by their relationship to simultaneous CF inputs^56–59^. To gain insight into the relationship between GrC and CF reward dynamics, we imaged CF activity (reported by Purkinje dendritic Ca^2+^ spiking) during push-for-DA (Fig. 5A, 11 mice). Sorting CFs by their activity just after self-stimulation delivery (spike rate [0.1, 0.2] s), we noted numerous DA-responsive neurons (Fig. 5B, 1,048 CFs). We classified CFs as DA-responsive if their mean spike rate [0.1, 0.2] s was >1.3 Hz (Fig. 5C, 66% of CFs). We noted that such responses were absent when reward was omitted. Having previously observed similar responses during a water reward task, we compared these DA responses to those in water in a subset of mice trained in both tasks (Fig. 5D, 8 mice). Water responses (Fig. 5E), though comparable to those we reported previously^16^, were far smaller than those seen for DA rewards. Quantitatively, water-responsive CFs by the above cutoff comprised 49% of cells, but their response spike probabilities were far lower than for DA (Fig. 5F). On a per-session basis, DA-responding and water-responding cells comprised a comparable 34±9% and 47±7% of CFs, respectively (Fig. 5G, p=0.8). By comparison, applying the same criteria to classify reach-responding cells identified only 9±2 and 6±2% of cells in DA and water sessions, respectively (Fig. 5H). Spike rates in response to DA (4.1±0.03 Hz) far exceeded the response to water (1.9±0.02 Hz; Fig. 5I), but peak response latencies were the same: 134±1 ms (Fig. 5J). Thus, many CFs responded rapidly following DA rewards with far higher probability than typical responses to water rewards.

**Extended Data Fig. 5 |:**
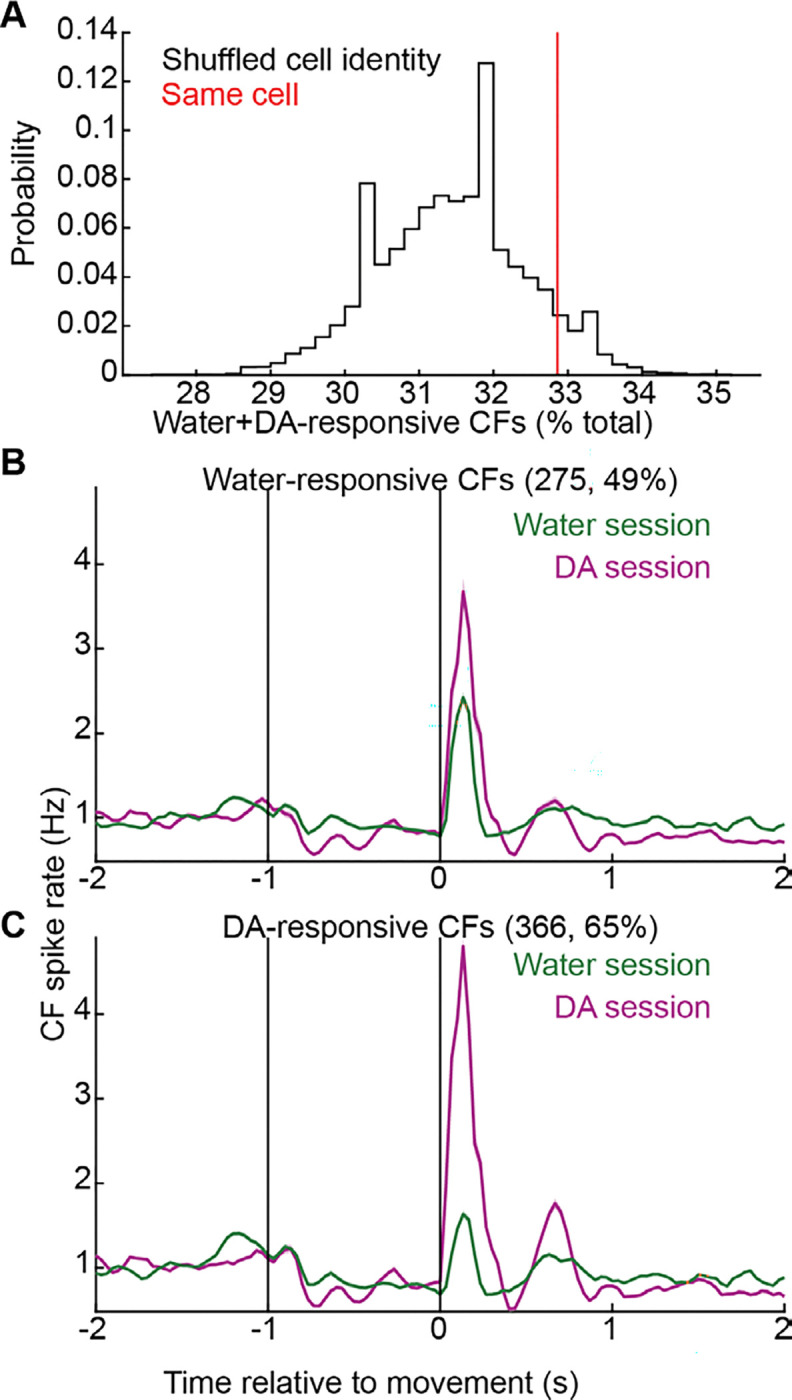
Related to Fig. 5 **A**, To shuffle-control the water+DA-responsive CF proportion in Fig. 5P, we performed 10,000 random shuffles of CF identity and computed the rate of “doubly responsive” CFs in these shuffled water/DA pairings, showing that the true data were not markedly higher than what would be predicted by chance, given the level of water and DA responsiveness in the population overall (p=0.06, permutation test). **B,C**, For CFs that responded to water (**B**) or to DA (**C**), traces show their responses recorded in both the water session and the DA session. Water-responsive CFs were even more responsive for DA, while DA-responsive CFs were much more weakly responsive to water, on average.

To determine whether individual CFs responded to both water and DA rewards, we registered Purkinje dendrite identities across tasks (Fig. 5K). Visually, we noted limited overlap when sorting CFs based on water responses (Fig. 5L,N) or DA responses (Fig. 5M,O). To compare each CF’s DA and water reward responses, we scattered each cell’s mean spike rate from [0.1,0.2] s from the two recordings (Fig. 5P). Roughly 1/3^rd^ of CFs cleared the same 1.3 Hz cutoff for both DA and water rewards (Fig. 5Q,R). However, in contrast to the far above-chance GrC generalization above, and likely due both to the famously high correlations among groups of CFs^60^ and the very high proportion of DA-responsive CFs overall, water+DA-responsive CFs were not substantially more common than in shuffle controls (p=0.06, Extended Data Fig. 5A). In addition, even CFs responsive to both water and DA responded far more strongly to DA (Fig. 5S,T, Extended Data Fig. 5B,C). Overall, DA elicited widespread spiking responses in CFs, which also often responded to water reward but more weakly.

### Naïve mice pushed at modest rates solely for delayed CF activation, with similar predictive GrC ramping

**Extended Data Fig. 6 |:**
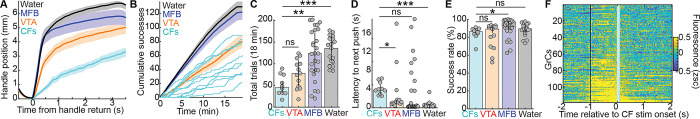
Related to Fig. 6 **A**, Handle position relative to automated return from the previous trial (influenced by both the speed to initiate the subsequent trial and the propensity to complete each attempted reach). **B-E**, To compare CF “rewards” to ‘true’ rewards, we computed the metrics from Fig. 6D–G week to push for a 50 ms 5 mW pulse of 640 nm light over a cerebellar cranial window (as for the CF-activation mice). For metrics from Fig. 6D–G, CF stim rewards were generally less rewarding than the other types, with a rough hierarchy of CF<VTA<MFB<Water (**C-E** p-values: 0.2; 0.003; 7×10^−5^; 10 CF, 13 VTA, 27 MFB, 19 Water sessions). **F**, For the subset of GrCs classified as ramping during push-for-CF stimulation (Fig. 6I), rasters show trial averages of the individual GrCs (n=118).

**Figure 6 |:**
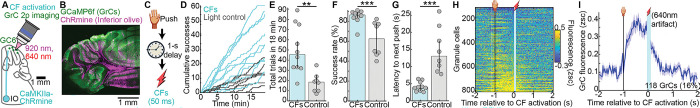
Naïve mice learned to push at modest rates solely for delayed CF activation, with similar GrC ramping **A-C**, CFs activated via ChRmine in inferior olive (IO) in GrC GCaMP transgenics. **(A)** Schematic. **(B)** Histological section showing GrC GCaMP and CF ChRmine expression. **(C)** Paradigm. CFs activated for 50 ms, 1 s after pushing, for a week (10 M1xztTAxGCaMP6f + IO-ChRmine mice; 4 no-opsin light control mice). **D-G**, Mice were moderately motivated for CF “rewards” in excess of light-only controls. **(D)** Cumulative successful pushing movements over time. Lines show individual sessions (10/10 opsin and 7/4 no-opsin sessions/mice). **(E)** Successful trials in 18 min. **(F)** Percent of pushes that were completed (>6 mm). **(G)** Latency from handle return to completion of the next push (**E-G** p-values: 0.003; 0.0009; 5×10^−5^). **H,I**, Neural activity during CF stim. **(H)** Rasters of all GrCs aligned to CF stim onset (812 GrCs from 10 sessions/mice). **(I)** GrCs that ramped prior to expected CF activation (16%, 118 delay-ramping GrCs as classified in Figure 2).

If CF reward feedback helps predict reward timing from GrC activity, might it also help *motivate* operant behavior? To test this, we trained naïve mice to push solely for delayed CF activation ‘rewards.’ We used GrC imaging mice (M1-Cre X ztTA X GC6f) injected with the red-shifted opsin ChRmine in the inferior olive to activate posterior cerebellar climbing fibers through an imaging cranial window (Fig. 6A,B; 10 mice; 4 no-opsin light control mice). To simulate the observed CF reward response, we delivered one 50-ms pulse of 640 nm light centered over Lobule VI triggered 1 s after successful reach end (Fig. 6C). After 1 week of training, mice executed 46±4 successful pushing movements in an 18 minute session (Fig. 6D,E; light control: 19±3), suggesting a surprising capacity of this pathway to drive behavioral reinforcement. Nonetheless, motivation under CF reinforcement was substantially lower than with true rewards (Extended Data Fig. 6). Higher performance in CF-motivated mice than light controls reflected both significantly higher proportions of pushes that were completed and more rapid initiation of the next trial following handle return (Fig. 6F,G), but latencies were nevertheless longer than for mice motivated with true rewards (Extended Data Fig. 6).

We next examined GrC responses during push-for-CF activation. Because the 640 nm light partly contaminated the green imaging channel, we discarded imaging data during the 50-ms pulse of CF activation light. We found that a small but notable group of GrCs appeared to show elevated activity during the delay period prior to CF stimulation (Fig. 6H). Examining this group of neurons in more detail, we found delay ramping activity similar to that seen in push-for-reward sessions (Fig. 6I, Extended Data Fig. 6F). Thus, reward-evoked CF activity can reinforce, if not suffice to fully motivate, associative operant behavior, while similarly eliciting GrC activity ramps that link the action and future stimulation outcome (**Discussion**).

### Intact granule cell delay period signaling is required for normal push-for-DA learning

**Extended Data Fig. 7 |:**
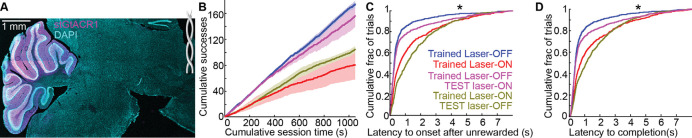
Related to Fig. 7 **A**, Histological image showing expression of stGtACR1 (YFP) in cerebellar GrCs and a slit in the tissue at the site of the MFB probe insertion. **B**, Analogous to Fig. 7G but showing averages across trials by condition (487, 538, 483, and 617 trials for trained laser-off, trained laser-on, trained laser-off/test laser-on, trained laser-on/test. 6 laser-ON and 5 laser-OFF mice). **C,D**, Analogous to Fig. 7H but showing latency specifically after unrewarded trials (**C**) (aborted or omission), or to movement completion for successful trials (**D**), trained off vs on both p<10^−6^.

**Figure 7 |:**
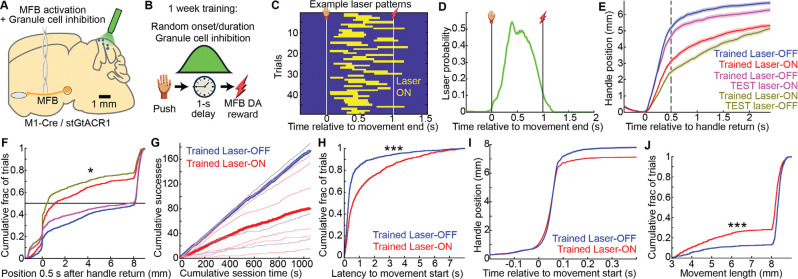
Perturbing delay-period GrC activity throughout training disrupts push-for-DA learning **A**, Strategy to inhibit GrCs using stGtACR1 while training push-for-MFB activation. **B**, Paradigm to inhibit GrCs during the delay period throughout learning (11 M1/stGtaCR1 mice; 6 laser-ON trained, 5 laser-OFF trained). **C,D**, Laser activations during example session (**C**) and all sessions (**D**, 2,785 trials from 33 sessions). **E**, Handle position aligned to its return from the previous trial (Trained laser-ON: 789 laser-on and 822 laser-off trials. Trained laser-OFF: 827 laser-off and 812 laser-on trials). Dashed line denotes quantification point for **F**. **F**, Cumulative histogram across trials of the handle position [0.45,0.55] s (n values from **E**; Bonferroni-corrected p-values: Trained Laser ON vs OFF, p<10^−6^; Trained Laser ON vs Test laser OFF p=10^−5^; Trained laser OFF vs test laser ON, p=0.01). **G**, Cumulative successful trials, thin lines are individual session and thick lines the medians. **H**, Cumulative histogram across trials of latency from handle return to movement onset (for trials with latencies <8 s, 723 laser-ON and 760 laser-OFF trials, p<10^−6^). **I**, Handle position aligned to movement onset. **J**, Cumulative histogram across trials of movement length (789 and 827 trials, p<10^−6^).

We next evaluated the causal role of action-DA interval GrC activity for associative learning. We used Math1-Cre X LSL-stGtACR1 mice to provide randomly timed optogenetic GrC silencing selectively during the delay period throughout push-for-MFB learning (Fig. 7A,B; Extended Data Fig. 7A). One group of M1xGtACR animals received a single pulse of light centered on Lobule VI during the delay period before MFB reward on every trial for 1 week. Pulses had random onset delay (340±3 ms after pushing >6 mm) and random duration (370±1 ms; Fig. 7C,D). A second group of littermate controls received normal (i.e., no-laser) training (6 laser-ON trained and 5 laser-OFF trained M1xGtACR mice). To evaluate motivation, we first aligned the handle motion to the time when it returned from the previous trial. If an animal consistently completed pushes immediately after the handle returned, this curve would quickly reach the maximum achievable extent (8-mm). Instead, laser-ON trained mice generated curves that rose much more slowly (Fig. 7E,F, p<10^−6^). As a result, laser-ON trained mice executed fewer successful movements (Fig. 7G, Extended Data Fig. 7B). This reflected two contributing factors: laser-ON trained mice (1) took longer to initiate trials following handle return (Fig. 7H, Extended Data Fig. 7C,D); and (2) made significantly shorter and more incomplete reaches (Fig. 7I,J). Finally, after the test day, we reversed the laser setting for the two groups: laser-ON trained mice performed with no laser, while laser-OFF trained mice performed with the laser on. Applying the laser slightly reduced the performance of laser-OFF trained mice, but removing the laser from laser-ON trained mice did not immediately improve performance, suggesting a longer-term effect on task learning (Fig. 7E,F). Thus, GrC delay period activity is required for normal push-for-MFB operant learning, comparable to the effect we previously observed when disrupting delay-period Purkinje cell activity during push-for-water learning^16^.

## DISCUSSION

### Broadening of cerebellar reward processing

By imaging cerebellar GrCs and CFs in mice pushing for delayed self-stimulation, we found that cerebellar neurons robustly predicted and responded to rewarding outcomes that were passively consumed. The cerebellum used a generalized strategy likely to be well-suited to predicting reward timing. GrCs generated temporally heterogeneous activity ramps that linked the action to the expected time of DA release, scaling their duration to match the action-DA interval when doubled from 1 to 2 s (Fig. 2). Most CFs generated time-locked spikes ~130 ms after self-stimulation onset (Fig. 5). GrC activity ramps thereby linked the action to future reward-evoked CF feedback, which could enable downstream neurons to generate well-timed extended activity ramps^16,29,61,62^. This representational strategy was preserved in individual mice sequentially trained across DA and water rewards, despite the far greater anticipatory and consummatory orofacial movements present in water reward sessions (Fig. 3, 5). In fact, many GrCs (Fig. 4) and CFs exhibited cross-activation in both water and DA contexts (Fig. 5). This suggests that some cells may encode more abstract features of rewarded behaviors common to both contexts. Causally, delayed CF activation on its own (in place of reward) drove weak associative operant behavior in naïve mice, also eliciting predictive GrC activity ramps (Fig. 6)—to our knowledge the first demonstration that cerebellar reward signals suffice to reinforce a novel action. Together these results suggest deeper integration of cerebellar circuits into reward prediction networks than previously understood.

### Computational role of cerebellar dopamine prediction signals

The temporal distribution of ascending and descending anticipatory GrC activity ramps forms a temporal “basis set”^63–67^ (Fig. 2W,X), which is central in cerebellar temporal prediction theories^68^. In this study, we demonstrated that GrC temporal basis sets present even for abstract events like self-stimulation rewards requiring no consummatory actions. The basis set had several other important features. First, “temporal scaling” enabled GrCs to span the interval from action to the expected time of self-stimulation reward, which is when CF feedback signals arrived. This is expected to make GrC ramps initiated seconds prior to reward still “visible” to CF-dependent plasticity mechanisms in Purkinje cells^69^. Second, many basis elements (individual GrCs) generalized between anticipation of DA and water reward despite their very different sensorimotor contexts. Similarly, many CFs spiked after both water and DA rewards, reminiscent of results in which CFs cross-activate to different events^46^ depending on salience^22,70^. These different forms of generalization (temporal scaling and cross-activation between reward types) may permit the cerebellum to compute general timing signals when knowledge of one context applies in another, or possibly even facilitate coordination in multimodal contexts^71–75^. At the same time, the large number of GrCs with distinct activity between contexts could support contextual disambiguation. Both generalized and distinctive GrC dynamics could be useful for different downstream computations.

Comparing the short versus long delay basis set, we found that descending GrC activity ramps were sometimes more common in 2-s delay imaging sessions than 1-s (Fig. 2). This could have a trivial technical explanation, for example, that reductions in activity are more difficult to detect with Ca^2+^ indicators^76^, such that deeper and more extended troughs in 2-s delays are more likely to be detected. Alternatively, it could be a biological adaptation, for example recruiting more GrCs to provide additional accuracy for timing estimates which are more difficult with ramping signals at longer durations^77^. Mechanistically, these GrC and CF dynamics likely rely on a variety of recurrent and amplificatory circuits^78–82^, local temporal patterning^83^, and interactions with the forebrain^29,84–86^. Overall, we propose that GrC temporal bases and CF reinforcement signals are generated to anticipate a broader set of salient events than solely those requiring physical actions.

### Behavioral contributions of cerebellar dopamine reward prediction

Replacing “reward” with CF activation sufficed to drive a weaker form of operant behavior that also elicited anticipatory GrC ramping (Fig. 6), while chronic perturbations of GrC delay activity disrupted push-for-DA learning (Fig. 7). Together these results clearly indicate causal cerebellar involvement in reward-driven associative learning, as previously shown in consummatory reward contexts^16,87^. Nevertheless, the *specific* role of the cerebellum *compared* to other brain networks like the nigrostriatal system remains unknown in action-reward associative learning. Delineating these comparative contributions will be essential to building a holistic brain-wide model of action-reward learning. Several possibilities could be explored.

First, the nigrostriatal system seems to be paramount in driving motivated behavior^88^. Our CF causal experiments suggest that the cerebellum may also reinforce self-initiated actions, but in a more limited way (Fig. 6 CFs vs VTA/MFB, and compared to cerebellar manipulation effects in motor learning studies^89–91^). Still, we cannot exclude that our study underestimates CF capacity to reinforce volitional behaviors: (1) our virus infected only very a small number of CFs (Extended Data Fig. 6A) (2) our cerebellar illumination was likely limited to, at best, ~ 1mm^3(92)^ (or ~2%^93^) of cerebellar volume; and (3) bulk optogenetic stimulation activates both the target reward-driven CFs as well as others. Furthermore, several GrC findings in this study bear on the question of motivation and cerebellar activation. VTA DA reward drove weaker anticipatory GrC signals than MFB reward (Extended Data Fig. 2), and perhaps not coincidentally also had lower motivation levels (Fig. 1). This could reflect (a) lower VTA motivation due to nonspecifically activating heterogeneous DA neurons^38^, not only those projecting to ventral striatum^94^; or (b) higher MFB motivation due to activation of other motivating but non-dopaminergic fibers^39–42^. CF-stimulation-driven operant behavior had even more rare GrC ramping signals, and again perhaps not coincidentally also drove even lower motivation levels (Fig. 6). A fascinating topic for future study would be the relationship between cerebellar reward prediction and motivation levels, ideally in a context where motivation can be systematically varied over trials in individual animals.

Second, our data along with many prior theories and studies suggest that GrC-CF-Purkinje cell microcircuits are likely especially well-suited to making precise computations of timing^61,95–98^, in this case reward timing. Thus, one possibility is that cerebellar timing estimates are fed downstream to refine action-reward learning in nigrostriatal networks^99,100^. Third, the high diversity of GrC information sources especially in lobule VI^86,101^ along with the astronomical GrC-Purkinje cell convergence ratio^102^ may enable cerebellar microcircuits to make exceedingly complex reward associations. Thus, the cerebellum may excel in identifying obscure reward associations and their timing, especially early in learning when fast cerebellar plasticity processes may be most dominant^103,104^—an ability that could benefit a host of associative learning processes elsewhere in the brain.

Overall, demonstrating that cerebellum both anticipates rewarded outcomes and suffices to reinforce the predictive action—even when no consumption movements are needed—may link it more strongly to broader brain reward networks and associated disorders, substantially broadening possible cerebellar contributions to reward-driven learning and behavior.

## Methods

### Experimental Model Details

#### Mice

Both male and female mice were used (13 male and 14 female).

All GrC imaging experiments used Math1-cre / Ai93 (TIGRE-LSL-TRE-GCaMP6f) / ztTA (R26-CAG-LSL-tTA) mice, aged 6–16 weeks, which selectively express GCaMP6f in cerebellar GrCs.^17^ GrC imaging experiments with optogenetic stimulation of ventral tegmental area dopaminergic neurons used DAT-Flpo / Math1-cre / Ai93 / ztTA mice, aged 6–16 weeks, which express flippase in dopaminergic neurons.^47^

Imaging CF activity in Purkinje dendrites used viral induction of PkC-specific R-CaMP2 in Math1-cre / Ai93 / ztTA mice with or without DAT-Flp, or PCP2/GCaMP8m mice with or without DAT-Flp. PCP2/GCaMP8m mice selectively express GCaMP8m^105^ in cerebellar PkCs.

Selectively inhibiting GrC activity employed Math1-Cre X stGtACR1 mice.

All procedures were approved by the NIH animal care and use committees.

### Method Details

#### Virus

In Math1-cre / Ai93 / ztTA mice, PkC-specific promoter (L7–6)^106^ was used to drive expression of the red Ca^2+^ indicator R-CaMP2,^107^ which was packaged into AAV-L7–6-R-CaMP2. Plasmids were generated by VectorBuilder and packaged into AAV9 by the National Eye Institute Ocular Gene Therapy Core. Virus was injected at ~10^12^ genomes/mL.

In DAT-Flpo mice, Flpo-dependent promoter (fDIO) was used to drive expression of the red-shifted opsin ChRimson^108^ in ventral tegmental dopaminergic neurons, transfected via AAV-Ef1a-fDIO-ChrimsonR-tdTomato. Virus was injected at ~10^12^ genomes/mL.

For CF activation studies, AAV-CaMKIIa-ChRmine-mScarlet-Kv2.1-WPRE was injected into the inferior olive to drive expression of opsin in cerebellar climbing fibers. Virus was injected at ~10^12^ genomes/mL.

#### General Surgical Procedures

We conducted surgical procedures as previously described.^16^ Mice were anesthetized via isoflurane (~2% in ~1L/min of O_2_) and secured in a stereotaxic device (Kopf). We cleaned the scalp and removed hair using depilatory cream. We removed a patch of skin with a mediolateral span from ear to ear, and a rostrocaudal span from the back of the ears to the eyes. We scraped the entire exposed skull surface free of soft tissue and then used VetBond (3M) adhesive to seal the edge of the skin incision to the skull.

For DAT-Flpo expressing Math1-cre / Ai93 / ztTA or PCP2/GCaMP8m mice, mice received an infusion of 500 nL at a rate of 100 nL/min of AAV1-ef1a-fDIO-ChrimsonR-tdTomato (Addgene, Addgene_171027) into the VTA with a ~25 um diameter-tip glass capillary using the following coordinates relative to bregma: AP, −3.4 mm; ML, +0.5 mm; DV, −4.3 mm. We waited 5 minutes post-infusion to withdraw the tip. A 5 mm length fiber optic cannula from ThorLABS (either CFML22L05 or CFMLC22L05, 0.22 NA, 0.2 mm diameter) was then implanted at the following coordinates relative to bregma: AP, −3.4 mm; ML, +0.5 mm; DV, −4.1 mm. The cannula was secured to the skull with Metabond (Parkell). Virus was allowed to express for 2 weeks prior to stimulation.

For mice used for the MFB experiments, a 5 mm length tungsten electrode (ProTech International, 8IMS303T3B01) dyed with DiD (Invitrogen, V22887) or FastGreen (Sigma Aldrich, F7252) dissolved in acetic acid was lowered into the MFB using the following coordinates relative to bregma: AP, −1.4 mm; ML, +1.2 mm; DV, − 4.8 mm. The electrode was then secured to the skull with Metabond.

To express R-CaMP2 in PkCs in Math1-cre / Ai93 / ztTA mice, we drilled a craniotomy 0.3 mm right of the midline and centered on the post-lambda suture. We inserted a ~25 μm diameter-tip glass capillary ~300 um below the pial surface and injected 500 nL of AAV-L7–6-R-CaMP2 into the tissue and waited 5 minutes after injection before withdrawing the tip. Virus was allowed to express for 2 weeks prior to imaging.

To express ChRmine in the inferior olive, the pipet tip was lowered at an azimuthal angle of 40 degrees, 0.5 mm left of the midline, and brought to the visual boundary between the bottom of the cerebellum (Lobule IX) and the top of the brainstem, and inserted 2.8 mm past the brain surface. 80nL was injected and then the pipet retracted 50 μm, to a maximum of 400nL volume and a minimum 2.6 mm insertion depth.

#### Histology

Mice were deeply anesthetized with isoflurane, and then transcardially perfused with 1x PBS followed by 10% formalin. The brain was then dissected out and left overnight in 10% formalin. Following overnight fixation, the brain was transferred to a 30% sucrose solution in 1x PBS until the brain sank to the bottom of the solution. We then embedded brains in OCT Compound (Fisher HealthCare) prior to sectioning. Coronal or sagittal sections (50 um) were collected using a Microm HM550 cryostat into well plates containing 1x PBS.

For GrC GCaMP6f histology, we performed immunostaining on sagittal sections by first washing 3 × 10 minutes with 0.1% PBS-T, then blocking in 10% NGS in PBS-T. We then stained the sections using rabbit anti-GFP (Abcam, AB290, 1:1500 dilution) for ~48h, followed by 3 × 10 minutes washes with 0.1% PBS-T, followed by Alexa 488 goat anti-rabbit (Abcam, AB150077) secondary antibody stainings for ~2 hours. For PkC GCaMP8 histology, we followed the same steps above except we used a 1:2000 dilution for rabbit anti-GFP.

For further histological processing of mice expressing DAT-Flpo, we performed immunostaining on coronal midbrain slices with 3 × 10 min washes of 0.1% PBS-T then 1h of blocking in 10% NGS in PBS-T. For coronal sections, we stained midbrain sections using chicken-TH (Abcam, AB76442, 1:2000) for ~48h at 4°C, follow by 3 × 10 min washes of 0.1% PBS-T, followed by Alexa 647 goat anti-chicken (Abcam, AB150083) secondary antibody staining for ~2 h.

We mounted all sections with DAPI mounting medium and imaged the slides using a confocal microscope with 405, 488, 561, and 647 nm lasers (Zeiss LSM 510). ChRimson opsin (tdTomato) is visible under 561 nm illumination without staining. For Math-1-Cre x stGtACR1 mice, stGtACR1 opsin (mScarlet) is visible under 561 nm illumination without staining.

For MFB histology, we directly mounted the sagittal sections in DAPI mounting medium and imaged using the 405 and 647 nm lasers.

#### Window and headplate implantation

Following viral injections and/or cannula implantation, we made the cranial window as previously described.^16^ We drilled a ~3.5 to 4 mm diameter cranial window centered over Lobule VI of the right vermis. We then used UV curing optical adhesive (ThorLABS NOA 81) to glue a 3 mm diameter glass cover slip onto a 3 mm outer diameter, 1 mm height, 2.7 mm inner diameter steel ring. We stereotaxically inserted the cured glass onto the cranial window site at a 15–20° angle counterclockwise from the sagittal axis and a 35–40° azimuthal angle from the vertical axis. We depressed the glass to a depth below the average depth of the underside of the skull at the window perimeter. We then sealed the steel ring to the skull using Metabond (Parkell).

We then put a custom headplate with a 5 mm central opening and two extensions with two holes for screws to head-fix mice onto fixation bars on the behavioral rig. We stereotaxically lowered the headplate, surface parallel to the glass window, onto the skull with the central opening centered on the window and then adhered the headplate onto the skull using Metabond (Parkell).

#### Behavioral Data Collection

Custom National Instruments cRIO software controlled and recorded the movement of a custom two-axis planar robotic manipulandum^43^. Through a single DAQ this device and software synchronously recorded: handle position; microscope frame counter TTLs; camera frame trigger TTLs; water solenoid and/or optogenetic laser and/or microwire stimulation TTLs. These signals were used to synchronize the behavior and imaging data post hoc and, in cases where dlc body tracking was performed, also the camera recordings.

#### Behavioral Training

During push-for-DA studies for either optogenetic stimulation of VTA-DANs or electrical stimulation of the MFB, mice were given ad libitum access to food and water. When mice were trained to push for water, mice were water restricted to receive 0.4 mL of water per every 10 g of free feeding weight to maintain 80% of their free feeding weight. Mice were weighed daily to monitor for excess weight loss in addition to checking for signs of lethargy, coat deterioration, hunching, and general distress. Mice obtained water until satiety during behavioral training; if this was insufficient, they received the remaining amount up to their dosage as supplementary water.

Push tasks employed our custom two axis robotic manipulandum.^43^ The push task had the following structure. Mice self-initiated trials by pushing the handle of the robotic arm. Trials were terminated when either of the two conditions were met: (1) animals reached the 8 mm end of the push length where the robot immediately terminated movement, or (2) animals ceased pushing the handle at any distance >3 mm for more than 100 ms. Following a delay at the end of a successful movement, reward was given. In push-for-VTA sessions, optogenetic stimulation was delivered by a 640 nm laser driver (OBIS) triggered by the cRIO to deliver light through a fiber optic cable coupled to the optic cannula targeting VTA. In push-for-MFB activation sessions, the cRIO trigger drove an electrical current through the tungsten electrode targeting MFB. For water reward, a water droplet was delivered in front of the animal’s mouth via a gravity-fed reservoir through a solenoid valve. The main imaging dataset had a 1-s-delay, while for the 1-s-to-2-s behavioral imaging studies the delays were 1-s or 2-s as indicated. Following an additional ~2-s delay, the robotic arm began automatically returning to the animal over the next 2 s, after which the mouse was able to self-initiate the subsequent trial.

For reward switching studies, we first trained mice on either push for stimulation or push for water without collecting images. After ~1 week of training, we imaged sample field(s)-of-view and then switched training to either push for water or push for stimulation respectively. After an additional ~1 week of training on the novel reward, we returned to the original field-of-view for imaging on the other reward, aligning neurons when possible.

For delay switching studies, we trained mice on push for stimulation with 1-s delay. After ~1 week of training, we imaged sample field(s)-of-view and then switched to training on a 2-s delay. After an additional ~1 week of training on the longer delay, we returned to the original field-of-view.

#### Two-photon microscopy

A custom two-photon microscope with mechanically articulating objective and both 920 nm (GCaMP) and remotely focused 1064 nm (RCaMP) excitation lasers was used for all imaging studies. GrC imaging used a 40× 0.8 NA Olympus objective while CF imaging for PCP2 X GCaMP8 mice used a 16× 0.8 NA Nikon objective. Remote focusing used an Optotune electrically tunable lens to manually pull the laser focus up from the GrC layer (where the 920 nm was focused) into the Purkinje dendritic layer (typically ~50–100 μm). Images were 512×512 pixels and frame rate 30 Hz. Chronic GrC imaging required angular alignment of the objective to the cranial window glass via red laser back reflection through an iris in the objective port; stage coordinates with respect to a “landmark” blood vessel; and finally manual alignment of the live image to the previous session’s mean image using fine motor and objective z-piezo positioning.

For imaging during fiber illumination of the VTA, an additional 625 nm short pass filter and stacked green or red emission filters on the respective PMTs were used to cut out 640 nm laser light bleed through. Since this was incomplete for the red channel, we also used post hoc temporal interpolation to remove remaining artifacts (Extended Data Fig. 1 for complete description).

For imaging during 640 nm activation of CFs through the simultaneous imaging objective, additional dichroic mirrors and substitutions were used to combine the 920 nm and 640 nm lasers and deliver them into the tissue while excluding them from the emission path. Due to the extended pulse duration (50 ms) which precluded the temporal interpolation strategy used for VTA activation, but conversely the much briefer total activation duration (also 50 ms), we simply discarded imaging data during those 2–3 frames due to high residual 640 nm laser bleed through into the green channel.

#### Granule cell optogenetic inhibition

For Math1-Cre X stGtACR1 studies, mice were randomly divided into two cohorts, with 6 mice in the laser-ON training group and 5 in the laser-OFF training group. Both cohorts were trained on the standard push-for-MFB activation task for a week. Laser-ON cohorts had an optical fiber positioned above the cranial window that delivered 5–10mW of 488 nm laser light with a random onset latency and duration following handle displacement exceeding 6 mm, triggered by the cRIO hardware. Onset latency after threshold-crossing, 340±3 ms, and duration 370±1 ms. After the week of training, both cohorts had one day with their laser condition “inverted”: laser-ON trained mice pushed for MFB activation with laser-OFF, while laser-OFF trained mice pushed with laser-ON.

#### Climbing fiber optogenetic activation

For CF activation studies, mice were trained for a week to push for a single 50 ms pulse of 640 nm light delivered either by optical fiber directly or coupled into a microscope objective positioned over the cranial window, delivered 1 s after movement completion. Control mice with cerebellar cranial windows but with no CF opsin were trained the same way.

#### Two-photon microscopy data preprocessing

All data was first motion-corrected using sequential large-displacement rigid correction followed by small-displacement nonrigid correction both provided by Norrmcorre^109^. Videos were subsequently corrected for slow drifts by dividing out exponential fits to the frame-averaged fluorescence across the recording session. GrCs were sorted using cNMF^1^. CFs were sorted using PCA/ICA^2^. In both cases, after automated sorting, we performed manual filter curation, splitting, and merging as needed. Final traces were obtained by back-applying the spatial filters to the movie and z-scoring each cell’s fluorescence trace and, for CFs, performing deconvolution and spike detection via thresholding and then spike rates in 150 ms windows.

### Quantification and Statistical Analysis

Throughout the text, quoted centers and ranges are median ± median absolute deviation of the sample (i.e., m.a.d./√n). All unpaired comparisons of two medians used the Mann Whitney U-test (Wilcoxon rank-sum test). All paired comparisons of two medians used the Wilcoxon sign rank test. All cases with multiple comparisons of two medians used Bonferroni correction of the raw p-values.

Data averaging throughout the text used several data alignment points. Cases where the data are said to be aligned to “movement endpoint” (or “movement” for short in some cases) referred to the last timepoint at which the handle displacement was <85% of its final position or, in cases where animals pushed very hard and exceeded the loose robotic “wall” at 8 mm displacement, the first time that the displacement exceeded 9 mm. Cases where the data are said to be aligned to “movement start” referred to the first time point following previous trial handle return that the speed (in 50 ms moving average windows) exceeded 55 mm / s. Cases where the data were said to be aligned to “reward” referred to the first timepoint at which either the water solenoid, or the MFB wire, or the VTA optogenetic laser trigger, received the first TTL rising edge.

## Extended Data

## Supplementary Material

Supplement 1 Video S1 | Push for VTA DA stimulation

Supplement 2 Video S2 | Push for MFB activation

Supplement 3 Video S3 | GrC GCaMP6f and Purkinje dendrite (CF) RCaMP2 imaging

Supplement 4 Video S4 | Purkinje dendrite (CF) GCaMP8m imaging

Supplement 5 Video S5 | 1-s and 2-s delay expert push for DA from the same mouse

Supplement 6 Video S6 | Push for DA and Push for water from the same mouse

## Figures and Tables

**Table:** 

REAGENT or RESOURCE	SOURCE	IDENTIFIER
**Antibodies**		
rabbit GFP	Abcam	AB290
chicken TH	Abcam	AB76442
Alexa 488 Goat anti-Rabbit	Abcam	AB150077
Alexa 647 Goat anti-Chicken	Abcam	AB150171
Alexa 647 Goat anti-Rabbit	Abcam	AB150083
**Viruses**		
AAV-L7-6-R-CaMP2	Inoue et al. and Nitta et al.	
AAV-ef1a-fDIO-ChrimsonR-tdTomato	Addgene	Cat #171027, RRID: Addgene_171027
AAV-CaMKIIa-ChRmine-mScarlet-Kv2.1-WPRE	Addgene	Cat #130991-AAV8, RRID:Addgene_130991
**Chemicals**		
C&B Metabond Quick Adhesive	Parkell	UN1247
DAPI Fluoromount-G	Southern Biotech	0100-20
Isoflurane	Henry Schein	CAS 26675-46-7
Vybrant DiD Cell-Labeling Solution	Invitrogen	V22887
FastGreen	Sigma Aldrich	F7252
**Mice**		
Mouse: Math1-Cre	Jackson Labs	Stock# 011104; RRID: IMSR_JAX:011104
Mouse: Ai93 (TITL-GCaMP6f)-D	Jackson Labs	Stock# 024103; RRID: IMSR_JAX:024103
Mouse: ztTA	Jackson Labs	Stock# 012266; RRID: IMSR_JAX:012266
Mouse: DAT-Flp	Jackson Labs	Stock# 035436; RRID: IMSR_JAX:035436
Mouse: PCP2-Cre	Jackson Labs	Stock# 004146; RRID: IMSR_JAX:004146
Mouse: TIGRE2-jGCaMP8m-IRES-tTA2-WPRE	Jackson Labs	Stock# 037718; RRID: IMSR_JAX:037718
Mouse: LSL-stGtACR1	Jackson Labs	Stock#: 037380; RRID:IMSR_JAX:037380
**Other**		
Fiber Optic Cannula, 0.22 NA	THORLabs	CFMLC22L05
2 Channel Electrode(MS303T/3-B/SPC)-Tungsten, 5 mm	Protech International	8IMS303T3B01
2 Channel Cable 305-000 No Spring	Protech International	305000XXXXCM001-35CM

## Data Availability

Code will be deposited at Github at time of publication, and data will be deposited at Dryad.
